# Washington on Dry Cupping

**Published:** 1853-08

**Authors:** A. H. Washington

**Affiliations:** Hannibal, Mo.


### SELECTED PAPERS.

### *Remarks on Dry Cupping.*

BY A. H. WASHINGTON, M. D.

I was called to a child, æt 7, (previously under charge of a "regular" physician and a steamer,) presenting the following symptoms, sequelæ of scarlet fever: pulse 140, remarkably hard and wiry; an abscess on one side of the throat as large as a turkey egg, and one on the other not quite so large; complete aphonia from condition of her throat; an abscess on back of left hand, left arm partially paralyzed; liver torpid, bowels irregular, sometimes costive, sometimes too loose, at that time costive; severe sciatic pain from left hip to the knee, could not bear anything heavier than a sheet; excessive emaciation as in the last stages of cholera infantum: heavy night sweats, but skin remarkably harsh and scaly during the paroxysm, and altogether presenting a most pitiable wreck of humanity; and her voiceless grief, as I inadvertently touched her sciatic leg, touched my heart deeper than the loudest groans.

I was fairly run aground, never had seen, heard, or read of such a case, and my only chance seemed to be, to do the best I could, and push off to Elkton, (Todd Co. Ky.,) and consult some experienced physician.

In my historical reading, a statement that Napolean's success was, in a great measure, owing to his bringing his forces from all quarters, and thus overwhelming the enemy, instead of endeavoring to succeed by one fierce charge, struck me with peculiar force; thus will I do, said I, when I commence practising. Some physicians arrange their forces incongruously, sometimes incompatibly, and then a helter skelter charge is made through the primæ viæ—merely to find themselves on the other side.

With too many, the one engrossing idea seems to be, to make a successful charge through the primæ viæ, and all other points are neglected; the skin for instance, is frequently entirely overlooked.

In pursuance of the plan of attacking the enemy from as many quarters as practicable, the following indications were laid down—1st, to reduce the pulse to the natural standard: 2d, to arouse the skin to a proper discharge of its functions: 3d, to regulate the liver and bowels: 4th, to cure the abscesses: 5th, to relieve the sciatic leg and paralyzed arm. From the history of the case, it was very evident that the condition of the pulse was the result of excessive stimulation, the brandy was therefore directed to be given in half the quantity and at double the interval: the whole body was to be sponged in warm water three times per day: a mustard plaster was applied the whole length of the spine: compresses (first applied cold, but found useless) wrung out of warm water were applied around her throat, and a strong vol. liniment was rubbed on the spine, after the mustard plaster had sufficiently drawn. On going to Elkton, was advised by a distinguished physician to stimulate with wine, or brandy, give a little quinine, and regulate the bowels by a grain or two of calomel, or blue mass: was told if her pulse was 120, she was bound to die. Fully believing she was "bound to die," concluded to omit the wine, as she was already over stimulated: calomel and quinine were administered as directed; the next day fever was high, and diarrhœa had followed the opening of the bowels: I quit all stimulants and purgatives with the determination to give nature as fair a chance as possible; had the patient put in warm bath twice per day, and continued the other remedies. In the course of five or six days, commenced dry cupping the whole length of the spine, instead of the vol. liniment and mustard previously used as a substitute: in the course of ten days, the patient had improved so much, I cautiously tried the mist. ferri. compos. and in less than four weeks from first visit, she was fat and rosy.

The deductions drawn from the case were few, but valuable ; hitherto I had only used dry cupping in strictly nervous cases, and it was to relieve the sciatica and paralysis of the arm, that it was tried; but finding the stomach completely digesting her food, the liver acting finely, and in consequence, the bowels becoming regular, the kidneys aroused to such free action as to relieve the skin, my eyes were opened to the fact, surprising to me, that dry cupping was a powerful alterative, and that the different organs could be effectually regulated through the medium of the nervous system. The anatomical structure of the nervous system gave me a clue to the rationale of the modus operandi of dry cupping: through the intimate connection of the spinal marrow with the whole system, when an impression is made on the spinal marrow, it is transmitted to the different organs through the respective nerves arising from it.

I had read much of nervous sympathy, but only a few isolated facts were found of any practical value, but that case was, to me, the "open sesame" of the nervous system; and its intimate connections were studied with far more pleasure from the fact that almost every item was of direct practical value.

Hence, for instance, if I find a pregnant female suffering with headache or cardialgia, I do not apply a mustard plaster or leaf of horseradish, nor administer alkalies, but I simply dry cup her spine, *break the chain of nervous sympathy*, and by regular repetition, accompanied with frequent sponging of the whole body, *keep it broken*, and unless she is excessively imprudent, no inconvenience is felt from her situation. Or if she is suffering from irritability of the bladder; from acrid urine, I calculate as certainly on a secretion of bland urine following dry cupping and sponging, as any other person would from the best diuretic known. Neither is there, to my mind, however it may appear to others, a tithe of the incongruity in recommending dry cupping and sponging for leucorrhea, amemorrhea, or their numerous sympathetic complications, that there is in the various plans recommended for those diseases, simply because the organ affected can be more easily reached and regulated through the nerves distributed to it, than by the indirect methods usually recommended. In conclusion, I will say, all I ask is a fair trial, and if any one finds my hobby does not ride well, let him stick to his own.

*Hannibal, Mo., March,* 1853.

